# Integrated multi-omics profiling uncovers the epigenetic, transcriptional, and metabolic landscape of prostate cancer progression

**DOI:** 10.1186/s12885-026-16232-7

**Published:** 2026-06-08

**Authors:** Christine Aaserød Pedersen, Maximilian Wess, Maria K. Andersen, Elise Midtbust, Abhibhav Sharma, Thomas Fleischer, Morten Beck Rye, May-Britt Tessem

**Affiliations:** 1 Department of Circulation and Medical Imaging, NTNU, Trondheim, Norway; 2 Clinic of Surgery, St.Olavs Hospital, Trondheim University Hospital, Trondheim, Norway; 3 Department of Cancer Genetics, Institute for Cancer Research, Oslo University Hospital, Oslo, Norway; 4 Department of Clinical and Molecular Medicine, NTNU, Trondheim, Norway; 5 Clinic of Laboratory Medicine, St.Olavs Hospital, Trondheim University Hospital, Trondheim, Norway; 6 BioCore - Bioinformatics Core Facility, NTNU – Norwegian University of Science and Technology, Trondheim, Norway; 7 ELIXIR Norway, NTNU – Norwegian University of Science and Technology, Trondheim, Norway

**Keywords:** Prostate cancer, Multi-omics, Multi-omics factor analysis framework (MOFA), Transcriptomics, Metabolomics, DNA methylation

## Abstract

A comprehensive understanding of the underlying molecular mechanisms of prostate cancer is essential for the development of precise diagnostic biomarkers. In this study, we applied the unsupervised multi-omics factor analysis framework (MOFA) to integrate DNA methylation, gene expression, and metabolic profiles derived from the same individuals, aiming to characterize the biological landscape of normal, malignant, and aggressive prostate tissue. Our analysis identified distinct molecular pathways associated with aggressive disease, specifically those involved in zinc metabolism, cell cycle regulation, smooth muscle architecture, immune activation, and tissue morphology. Key metabolites within the TCA cycle, amino acid metabolism, and lipid pathways were central to these signatures. Furthermore, we observed a consistent co-enrichment of SP1 and CTCFL binding regions among factor-associated CpGs, suggesting a model of global epigenetic reprogramming. These findings indicate a novel interplay between Polycomb deregulation, CTCFL-mediated chromatin remodeling, and SP1-driven transcriptional activation in shaping the prostate cancer epigenome. Apart from immune activation, the identified molecular signatures were validated in the TCGA cohort and demonstrated significant predictive value for disease recurrence. Overall, these results underscore the power of multi-omics integration in providing a holistic understanding of prostate cancer biology and its potential for clinical translation into prognostic biomarkers.

**Supplementary Information:**

The online version contains supplementary material available at 10.1186/s12885-026-16232-7.

## Introduction

Prostate cancer is one of the most prevalent cancers globally, affecting approximately one in eight men [1, 2]. The current diagnostic pathway struggles to accurately differentiate between patients with indolent and aggressive disease. This clinical challenge necessitates improved stratification methods to minimize unnecessary treatment side effects for indolent cases and ensure early intervention for aggressive cases.

Understanding which pathways lead to aggressive prostate cancer is needed for identifying biomarkers and drug targets. However, newer tissue-based molecular biomarker tools have shown inconsistent performance due to molecular and tissue heterogeneity [3]. Multi-omics analysis, which examines multiple omics layers simultaneously from the same samples, may elucidate complex mechanisms and molecular interactions, providing a deeper understanding of disease progression. Studies have shown that combining features from multiple omics layers enhances biomarker precision compared to single-omics approaches [4, 5].

This study utilized multi-omics factor analysis framework (MOFA), a versatile unsupervised machine learning tool that integrates various omics datasets into factors representing patterns of biological variation [6]. We included integrated metabolomics, transcriptomics and DNA methylation data into the MOFA model. Metabolites are integral to biochemical pathways and closely linked to cell phenotype, while transcriptomics data reflect gene activity and regulation. Lastly, the DNA methylation data covers important epigenetic modification crucial for regulating gene expression. Together, these three omics levels can shed a unique light on disease development at the cellular level.

The role of DNA methylation in gene expression and cell differentiation is complex. DNA methylation regulates chromatin structure by inhibiting or recruiting protein complexes that affect chromatin packing. One such complex is the polycomb repressive complex (PRC), which is implicated in prostate cancer aggressiveness and regulates cell differentiation through histone modifications, impacting chromatin accessibility for transcription [7, 8]. DNA methylation also influences transcription factor affinity to DNA and the transcriptional machinery’s affinity to genes, of which the most common and most studied is promoter methylation. Cell metabolism is also closely linked to methylation, e.g., by regulating the availability of methyl groups required for methylation [9]. Given the complexity and tissue-specific nature of these processes, studying various aspects of DNA methylation alongside gene expression and metabolites is essential for understanding these intricate biological mechanisms.

Prostate cancer research faces major challenges due to combined tissue and molecular heterogeneity, making it difficult to pinpoint the molecular mechanisms involved in aggressive disease. Comprehensive research using tissue from different prostate locations and integrating information across molecular layers is required to understand these mechanisms. Our study aims to capture the molecular complexity of tissue heterogeneity across omics levels and assess the predictive value of integrated multi-omics analysis of methylation, gene expression, and metabolites for cancer aggressiveness.

## Materials and methods

We included in total 280 samples from 57 patients. All patients underwent radical prostatectomy (RP) as curative treatment for prostate cancer at St Olavs Hospital, Trondheim, Norway, between 2007 and 2017. Immediately after RP, a 2 mm thick slice from the middle of the prostate was collected, frozen and stored at -80 °C by Biobank1, the research biobank of mid-Norway following the protocol established by Bertilsson et al. [10]. All patients delivered a written informed consent, and the study was approved by the Norwegian regional ethical committee (2017/576), which follows EU and Norwegian ethical regulations.

**Fig. 1:**
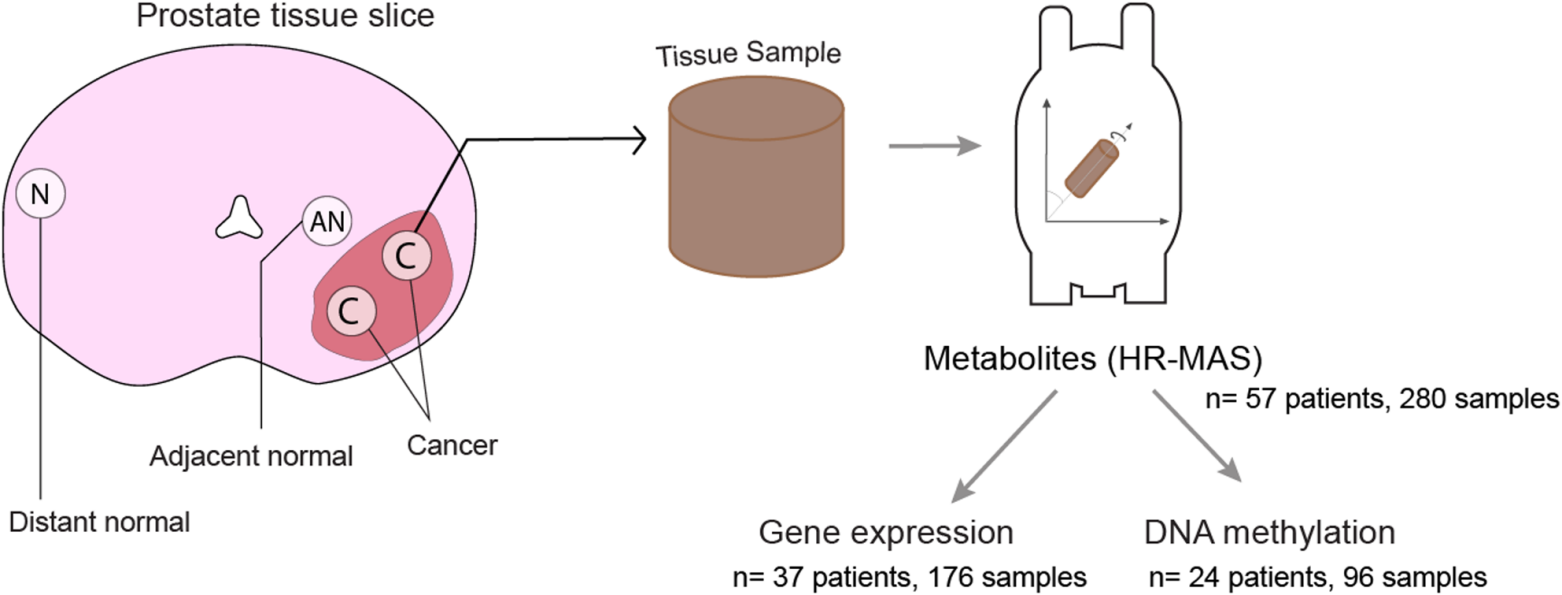
Sample collection and analysis. From each patient, 3–5 samples were collected. One sample was extracted far away from the tumor, one normal sample adjacent to the tumor, and two cancer samples were collected from the tumor area. The samples were drilled from the fresh frozen tissue slice and resembled cylinders in shape. The samples were analyzed with HRMAS (High-Resolution Magic Angle Spinning) for metabolite detection. Then DNA and RNA were extracted and analyzed for gene expression and DNA methylation, respectively

Three to five samples of 3 mm in diameter were collected from each frozen prostate slice, aiming to sample both tumor, normal and normal adjacent tissue, as shown in Fig. 1. After collection, the tissue type (normal or cancer) and eventual cancer grade was annotated by a histopathologist using a hematoxylin, erythrosine, saffron (HES)-stained section from the sample. Stroma and cancer content were estimated based on histopathology. Clinical follow-up data was collected 6–16 years after radical prostatectomy. Biochemical recurrence was defined as prostate-specific antigen (PSA) above 0.2ng/ml after radical prostatectomy. In the “recurrence” patient group (*N* = 45), we also included patients that had persistent PSA after surgery (PSA > 0.1ng/ml), meaning the PSA remained detectable. Patients in the “metastasis” group (*N* = 29) were diagnosed with metastatic disease by clinical imaging during follow-up, and patients in the “castrate resistant prostate cancer” group (*N* = 13) had elevated PSA levels after receiving androgen deprivation treatment after radical prostatectomy. The median follow-up time was 9.2 years (IQR 8.25–12.3 years). Sample characteristics are described in Table 1.

**Table 1:**
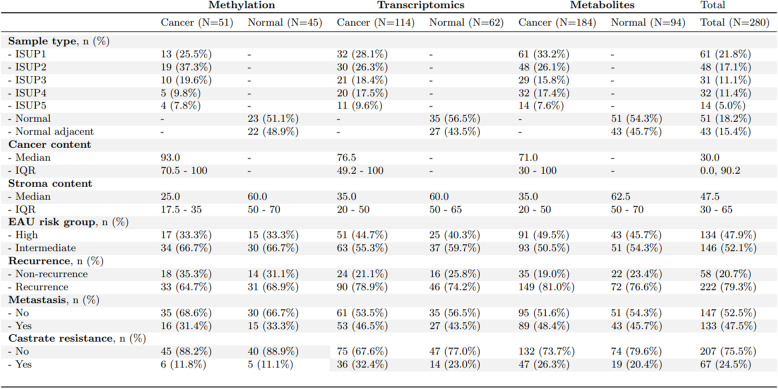
Sample characteristics for methylation, transcriptomics and metabolomics. IQR: interquartile range

### HRMAS NMR/metabolite data

All samples were first analyzed by HRMAS (high resolution - magic angle spinning), an NMR (nuclear magnetic resonance) spectroscopy method leaving the tissue intact for further analysis. It was performed on a 600 MHz Bruker Avance DRX600 (14.1 T) III NMR spectrometer (Bruker BioSpin, Germany) using the pulse sequence *noesygppr1d* on a ^1^H/^13^C MAS probe.

The raw spectra were preprocessed using Python version 3.8. After Automics phasing [11], the water peak visible from to 4.6ppm to 6ppm was removed, spectra were baseline corrected using asymmetric least squares smoothing [12] and aligned using icoshift [13]. Further, the peaks were mean matrix normalized. Peaks were picked and identified manually based on previous knowledge of HRMAS metabolites in prostate tissue [14–16], resulting in 30 identified metabolites. We performed relative quantification by integrating the area of the selected peaks. In case of several peaks per metabolite, we used the one that had the least overlap with other compound peaks. Two samples were removed during preprocessing due to noisy spectra because of high lipid levels, leaving 278 samples for analysis.

### Transcriptomics data

DNA and RNA was extracted from the tissue after HRMAS analysis, using the QIAGEN AllPrep DNA/RNA/miRNA kit. RNA from 176 samples was sequenced using the cDNA library SENSE mRNA-Seq Library Prep Kit V2 (600 ng RNA as input). Single-read sequencing was performed using the NextSeq 500/550 High Output kit v2.5 (75 cycles) on an Illumina NextSeq 500 instrument.

Produced FASTQ files were filtered and trimmed using fastp v0.20.0. The sequence alignment was performed using the STAR tool against the reference genome set GRCh38 release 92 (Ensembl). Subsequently, *featureCounts* [17] was used to extract gene counts from sequence reads according to the reference set.

RNA was sequenced in 4 different batches on different dates, and a batch effect was identified in exploratory analyses. The batch effect was adjusted for using ComBat-seq [18]. Further, the data was normalized for library size, and CPM (counts per million) values were extracted and log transformed.

### DNA methylation preprocessing

The isolated DNA from 96 samples was bisulfite converted using the EZ DNAMethylation kit, and the converted DNA was analyzed for methylation using the Illumina HumanMethylation EPIC BeadChip. For 64 of the samples, we used the Illumina EPIC array, and for 32 samples we used the EPIC v2 array.

We filtered out the probes with a detection *p* value < 0.05 and a bead count of under 3 in more than 5% of samples. Probes not measuring a CpG were removed, as were probes where the CpGs were closer less than 5 base pairs away from known SNPs, to avoid potential confounding by genetic variation [19].

Raw DNA methylation data was preprocessed using the R-package *minfi* and the funnorm-function was used for functional normalization [20]. The EPIC v1 and EPICv2 data was combined into one dataset including only the probes with matching probe sequence. A batch effect was observed between samples from the two arrays, that was corrected using combat [21]. We used methylation M values as input in the analysis and beta values for plotting, as recommended by Du et al. [22].

### Multiomics factor analysis

For multi-omics analysis, we used the Multi-Omics Factor Analysis **(**MOFA) tool [6] which is a an unsupervised machine learning model based on factor analysis tailored for handling multi-omics data when the omics levels are analyzed in the same sample. We used the whole metabolite data set (278 samples, 30 metabolites), the top 5000 variable genes and top 5000 variable CpGs from the transcriptomics dataset (176 samples) and methylation dataset (96 samples) based on the package authors recommendation (https://biofam.github.io/MOFA2/faq.html). We trained the MOFA model for 10 factors, since more factors did not increase the explained variance of the model (Supplementary Fig. 1). The MOFA model assumes linear mappings from latent factors to observed molecular measurements [6].

### Gene set enrichment

Using the MOFA package built-in function, we performed gene set enrichment analysis (GSEA) for each of the Factors 1–5 once using the positive weighted genes and once using the negative weighted genes. We used the gene sets from msigdb c5 (gene ontology) and extracted the top 5 GO terms with *p* < 0.01 (fdr) for the positive and negative weights for all factors. Further, GO terms were manually grouped together to reflect biological themes.

### Chromatin state assessment

To explore how the CpGs are related to chromatin states in prostate cancer tissue, we used ChromHMM data from the prostate cancer cell lines PC3 and LNCaP. ChromHMM is a software for learning and characterizing chromatin states to functionally annotate the genome by using a multivariate Hidden Markov Model (HMM) for identifying combinatorial patterns of histone marks obtained from ChIP-seq data [23]. ChromHMM segmentation data were downloaded from the ENCODE data portal [24] from two prostate cancer cell lines: PC-3 (Accession ENCSR103ZFL) and LNCaP (Accession ENCSR072ZGM). Functional regions were intersected with CpG positions using BEDTools v2.27.1 [25].

Enrichment of CpGs in a ChromHMM defined functional region was measured as the ratio between the frequency of the top (positive) quartile and bottom (negative) quartile of CpGs in each factor over the frequency of CpGs from the Illumina HumanMethylationEPIC array found within the same region. P-values were obtained by hypergeometric testing with the Illumina EPIC array probes as background. Multiple testing was accounted for using Benjamini-Hochberg correction.

### Enrichment for transcription factor binding regions

Maps of direct transcription factor-DNA (TF-DNA) interactions were obtained from the UniBind database [26]. The UniBind TFBSs (transcription factor binding sites) represent high-confidence direct TF-DNA interactions determined experimentally through ChIP-seq and computationally through position weight matrices (PWMs) from JASPAR [27]. The predicted transcription factor binding sites (TFBSs) were derived from 4114 ChIP-seq experiments for 268 TFs across cell types and tissues and were predicted to have high PWM scores and be near ChIP-seq peaks. TFBSs were extended by ± 150 bp (then called transcription factor binding regions; TFBRs) and were intersected with CpG positions using BEDTools v2.27 [25]. Since UniBind maps of TF binding sites are often derived from several ChIP-seq experiments for each TF, we merged the TF binding sites for all ChIP-seq experiments for each TF. Enrichment of CpGs in TFBRs was imputed using hypergeometric testing (R function phyper) using the IlluminaMethylationEPIC CpGs as background. Multiple testing was accounted for using Benjamini-Hochberg correction.

### Gene signature construction and validation of prognostic impact

**Fig. 2:**
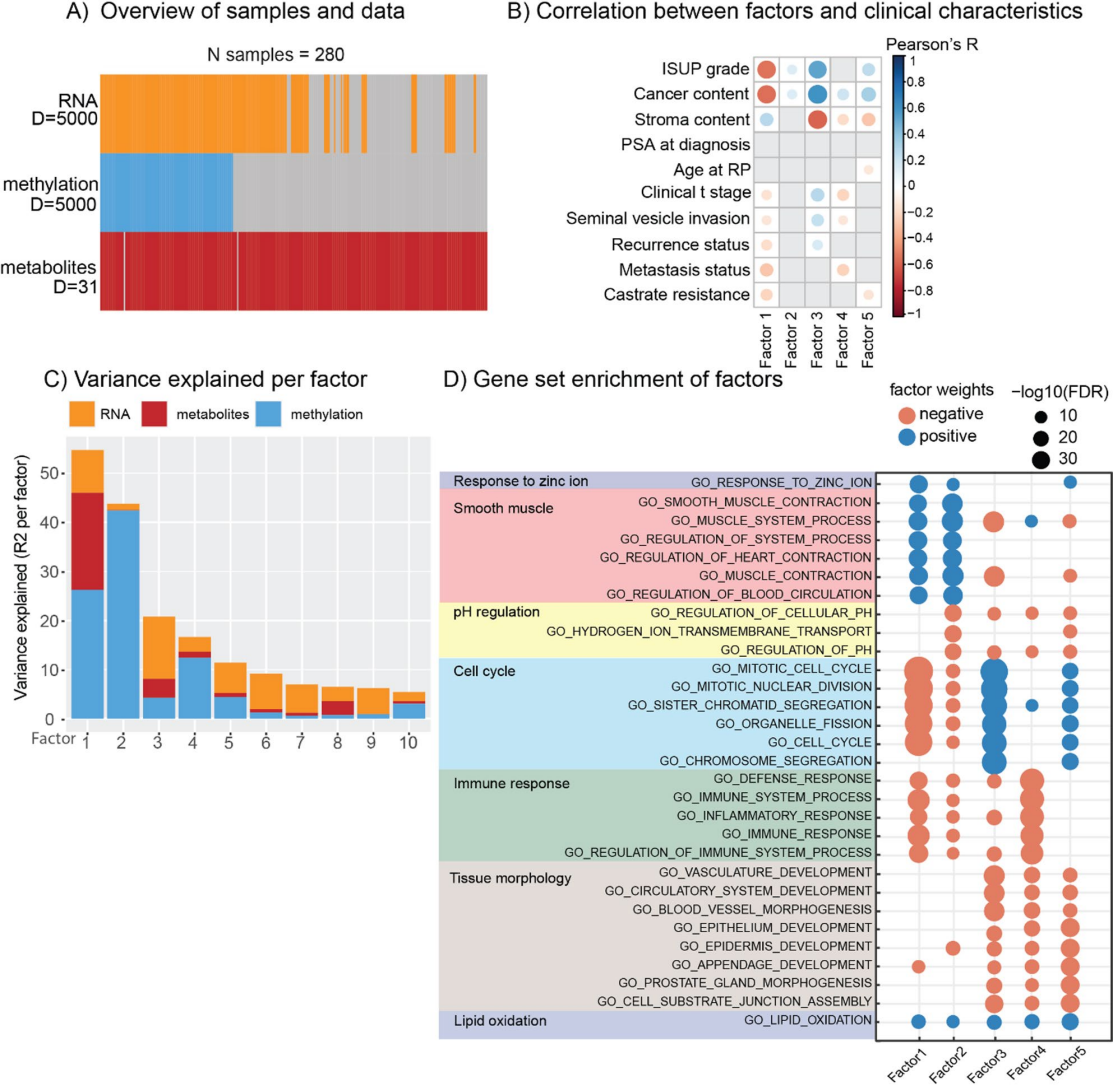
MOFA model reveals biology associated with tissue morphology and clinical outcome.** A** We included a total of 280 samples, incorporating 5000 genes (RNA), 5000 CpGs (methylation), and 31 metabolites into the model. Gray cells indicate that an omics type was not available for a patient. **B** Correlation between factors, and clinical and sample characteristics show that the factors are both correlated to sample characteristics such as ISUP grade and stroma content, but also clinical outcomes such as recurrence, metastasis and castrate resistance. **C** Contribution of each -omic level to the variance explained by the factors generated by MOFA. **D** Gene set enrichment of the genes in each factor, positive and negative weights analyzed separately. Several biological themes emerged, including response to zinc ion, smooth muscle, pH regulation, cell cycle, immune response, tissue morphology, and lipid oxidation. MOFA = Multi-Omics Factor Analysis; ISUP = International Society for Urological Pathology; PSA = Prostate-specific Antigen; RP = Radical Prostatectomy; FDR = False Discovery Rate

We constructed gene signatures for each of 5 biological themes (cell cycle, metallothionein, immune response, tissue morphology and smooth muscle; Fig. 2). For a given theme, we selected genes that were involved in the theme’s GO-terms and in the upper or lower quartile of the density distribution of weights in the MOFA factors associated with the theme.

To evaluate the prognostic relevance of gene signatures, we performed single-sample gene set enrichment analysis (ssGSEA; GSVA package) [28] using bulk RNA-seq data from TCGA-PRAD. The bulk RNA-seq expression data were obtained from cbioportal database (https://www.cbioportal.org/datasets) under the heading Prostate Adenocarcinoma (TCGA, PanCancer Atlas). Clinical metadata for TCGA-PRAD (portal.gdc.cancer.gov/projects/TCGA-PRAD) cohort were curated to construct a harmonized metadata table. The status of biochemical recurrence (BCR) was derived from the NEW_TUMOR_EVENT_AFTER_INITIAL_TREATMENT field, with entries labeled “Yes”, cases annotated as “1:Recurred/Progressed” in DFS_STATUS, or “1:DEAD WITH TUMOR” in DSS_STATUS. The resulting metadata were merged with the bulk TCGA-PRAD RNA-seq data and used for Kaplan-Meier and log-rank survival analyses to evaluate the impact of gene profiles on BCR. Each patient received an enrichment score per signature, which was stratified into low, medium, and high categories (low < 30%, high > 70%, medium 30–70% quantiles). Recurrence-free survival was then analyzed using Kaplan-Meier and log-rank tests across signature-defined groups, visualized using *survminer* in R [29]. 

### Feature selection and statistical analysis

To focus on the most dominant patterns in MOFA’s computed factors, we selected genes from GO terms that also had factor weights in the upper or lower quartile of the density distribution of the involved factor’s weights for each biological theme. Some GO terms contain large sets of genes, many of which overlap with other GO terms. For our analyses, we concentrated on genes with functions most relevant to the prostate, so only this selected subset is shown rather than all genes associated with each term. Similarly, we selected metabolites if their associated factor weight was in the upper or lower quantile of the density distribution of the involved factor’s weights. To describe the relationship of the various omics layers, we further selected genes, metabolites, enriched chromatin states, and TFBSs, where genes were significantly correlated (absolute Spearman’s rho > 0.45) with any other omics measurement. Further, we assessed whether expression levels of multi-omics data show clinical significance using Spearman correlation.

## Results and discussion

### Multi-omics factor analysis reveals biological themes associated with clinical outcomes

To reveal the intricate connections between transcriptomics, metabolomics, and epigenomics data, we used MOFA, an unsupervised factor analysis framework for multi-omics data. We used the 5000 most variable genes, 5000 most variable CpGs, and 31 metabolites from 280 samples (*N* = 57 patients) to train a model with 10 factors (Fig. 2A). Each trained factor is associated with the weight for each gene, metabolite, and CpG. A high positive weight reflects a strong association between a variable (i.e., a gene, metabolite, or CpG) and a factor, while a high negative weight reflects a strong inverse relationship. The trained model explained 76% of the total variance in methylation, 50% of the RNA, and 29% of the variance in metabolites (*R*²).

The factors of the MOFA model captured various aspects of prostate cancer-specific clinical and morphological variation (Fig. 2B), such as stroma and cancer content in Factors 1,3 and 5, and ISUP grade in Factors 1 and 3. Features associated with prostate cancer aggressiveness, castrate resistance, and metastasis status were captured in Factors 1, 3, 4 and 5. We chose to further explore the first 5 factors because they explained the most variance (Fig. 2C) and their strong correlations with clinical features (Fig. 2B, Supplementary Fig. 2).

To gain an understanding of the biological mechanisms underlying each factor, we performed GSEA separately on genes with positive and negative weights in each factor (Fig. 2D). GSEA revealed seven biological themes that emerged as sources of variation based on the first five factors (Fig. 2D). As this analysis focuses on exploring the relationship between various omics, we excluded the biological themes “lipid oxidation” and “pH regulation” due to a lack of significant correlations (Spearman’s *ρ* > 0.45) with either metabolomics or epigenomics measurements, resulting in five biological themes for further exploration (Fig. 2D): Response to zinc ion, smooth muscle, cell cycle, immune response, and tissue morphogenesis.

To further explore biological themes, we focused on genes and metabolites with the highest absolute weights in factors associated with each theme (Fig. 2D, Supplementary Fig. 3). For DNA methylation, we selected CpGs with positive weights in the highest quartile and negative weights in the lowest quartile to perform enrichment analysis for chromatin states (from prostate cancer cell lines LNCaP and PC3) and transcription factor binding regions, to assess whether CpGs are involved in regulatory areas of the genome.

### Loss of metallothionein expression, citrate and polyamine levels are associated with loss of normal prostatic functions in cancer development

**Fig. 3:**
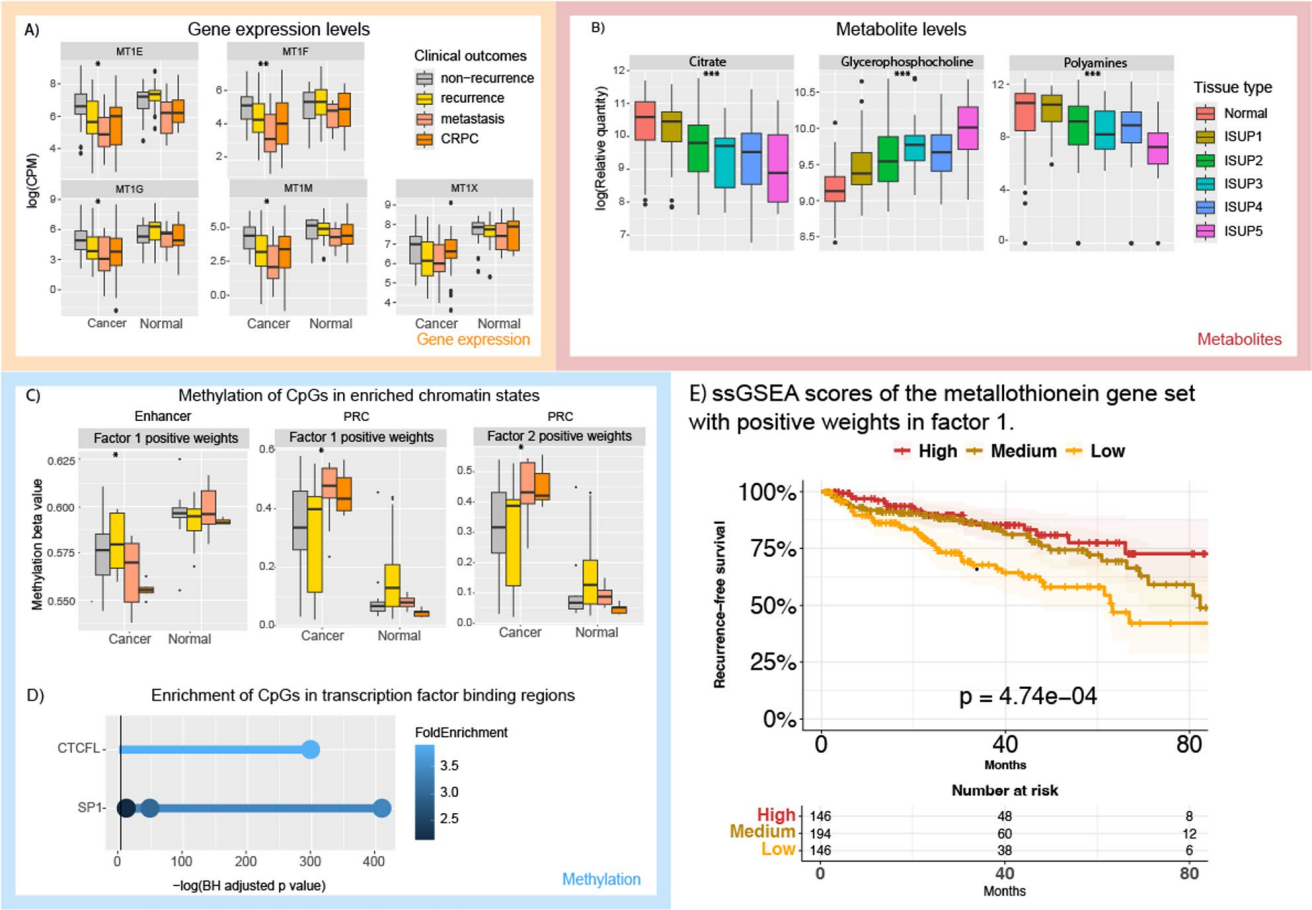
Response to zinc ion **A**) The metallothionein gene family (MT1E, MT1F, MT1G, MT1M, MT1X) shows a trend of decreasing expression in samples with recurrence, metastasis and castrate resistance (CRPC) compared to non-recurrence samples. **B** Citrate and polyamine levels, which have high positive weights in Factors 1 and 2, also show a trend of reduced levels in higher ISUP grades. While glycerophosphocholine shows increasing levels with ISUP grades. **C** Methylation of CpGs in enhancers shows increased methylation in cancer samples compared to normal, and a trend towards higher methylation in recurrence, metastasis and CRPC compared to non-recurrence (Factor 2 positive weights). The same pattern is observed in polycomb repressive complex (PRC) for Factor 2 positive weights. **D** CpGs with high positive weights in Factors 1, 2, and 5 are enriched in transcription factor binding regions of CTCFL and SP1. **E** The ssGSEA scores of the metallothionein gene set were significantly different when separating into high, low and medium risk for recurrence-free survival in the TCGA validation cohort. * = *P*-value < 0.5; ** = *P*-value < 0.1; *** = *P*-value < 0.01.

In Factors 1, 2 and 5, genes that were associated with the gene ontology term “the response to zinc ion” were enriched (Fig. 3). The metallothionein gene family (MT1E, MT1F, MT1G, MT1M, MT1X), an important gene family in the “response to zinc ion” GO term, showed a trend of decreasing expression in samples with recurrence, metastasis and castrate resistance (CRPC) compared to non-recurrence samples both in cancer and normal tissue samples (Fig. 3A). Metallothioneins can bind and transport zinc, an important metal in the normal prostate [30]. High metallothionein levels lead to zinc accumulation, which further inhibits the TCA cycle, causing an accumulation of zinc and citrate in normal prostate cells, which is then secreted as key components of the prostatic fluid [31]. Together with another prostatic fluid component, the polyamines, citrate and the polyamines showed a trend of lower levels in samples with increasing ISUP scores in Fig. 3B. This coincides with what has been thoroughly studied and presented in previous metabolomics studies by our group [14]. The lower expression of metallothionein genes may be the cause of less zinc transported into the mitochondria that again lead to higher consumption of citrate due to less inhibition of the TCA cycle [31]. In previous studies, we have associated lower zinc levels with higher ISUP grades [31] and lower citrate levels [32]. Both reduced citrate and the polyamine levels, which we found to be highly correlated [33], have previously been connected to aggressive prostate cancer and recurrence [16].

The CpGs with positive weights in Factors 1,2 and 5 were enriched for enhancer and PRC chromatin (Supplementary Fig. 4) and with transcription factor binding regions of CTCFL and SP1 (Fig. 3C and D). Enhancer CpGs in F1 with positive weights showed lower methylation in cancer compared to normal, while CpGs in PRC regions in F1 and F2 showed increased methylation in cancer and in more aggressive outcomes (Fig. 3C). Enhancer methylation is often associated with gene repression [34], suggesting that reduced metallothionein expression may be partly due to epigenetic regulation. SP1 is linked to prostate cancer aggression in several ways through both proliferation [35] and recently through induced epithelial-mesenchymal transition (EMT) in CTCs of prostate cancer [36, 37]. CTCFL is a paralog of the chromatin architectural protein CTCF and has been shown to aberrantly reorganize chromatin and to regulate AR in prostate cancer [38, 39].

To validate our gene expression metallothionein findings, we separated the ssGSEA scores of the metallothionein gene set into high, low and medium risk for recurrence-free survival in the TCGA validation cohort (Fig. 3E, Supplementary Figs. 5,6). Using 658 patients with up to 80 months of follow up, we found highly significant separation between the three risk groups (*p* = 0.0004). To conclude, we found and validated decreasing metallothionein expression and higher methylation with prostate cancers aggressiveness, while levels for citrate and polyamines decreased with higher sample ISUP grades. We hypothesize that metallothionein expression influences citrate levels through zinc binding and that metallothionein expression might be regulated by methylation and chromatin reorganization. These results demonstrate how metabolism, gene expression, and methylation might be interconnected in regulating citrate and zinc metabolism during the transition from normal prostate epithelial function to cancer development.

### The mitotic spindle checkpoint is associated with sample cancer content and lipid metabolism

**Fig. 4:**
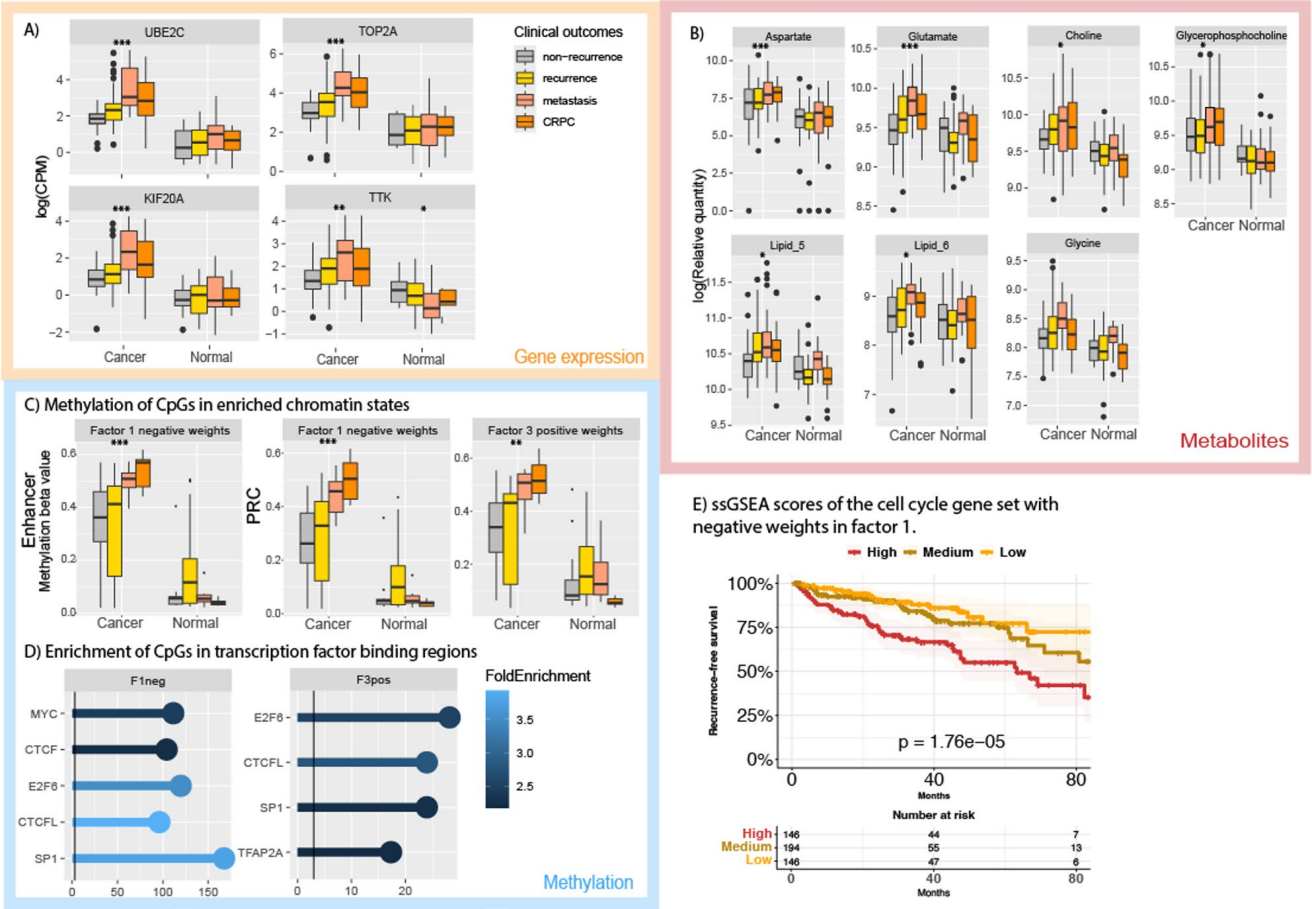
Cell cycle. **A** Gene expression shows a trend of higher expression in cancerous samples compared to samples with non-cancerous tissue. **B** Metabolites related to lipid and choline metabolism show a strong correlation with genes associated with cell cycle progression. **C** The CpGs were enriched for polycomb repressive complex (PRC) and enhancer chromatin states (Supplementary Fig. 4), exhibiting a higher methylation in cancerous samples than normal samples. **D** CpGs were also enriched in transcription factor binding regions associated with cell proliferation. **E** Validation of gene signature based on MOFA factor weights. * = *P*-value < 0.5; ** = *P*-value < 0.1; *** *P*-value < 0.01

GO terms related to cell cycle were enriched with negative weights in Factors 1 and 2, as well as positive weights in Factors 3 and 5, though only genes from Factors 1 and 2 were remaining after quantile filtering. These genes exhibit increased expression in cancer samples compared to normal samples (Fig. 4A) and are associated with various components of the mitotic spindle, including the centromere (TTK) and the anaphase-promoting complex (KIF20A, UBE2C, and TOP2A) [40, 41]. TDRD1 has been identified as a direct target gene of ERG overexpression in primary prostate cancer. The mitotic spindle checkpoint ensures that cells do not divide until all chromosomes are properly aligned. In cancer, this checkpoint may become disrupted, allowing cells to proceed with division even if chromosomes are not correctly aligned [42, 43]. This disruption may lead to accelerated cell cycle progression, chromosomal instability, and aneuploidy, all of which are commonly associated with cancer in general as well as prostate cancer [44, 45].

The genes further show a strong positive correlation with several metabolites which have negative weights in Factor 1 and positive weights in Factors 3 and 5 (Supplementary Fig. 7) All of these metabolites had higher levels in cancer samples compared to normal (Fig. 4B). Glutamate, aspartate, and glycine are involved in glycolysis and augmented amino acid metabolism, which are tightly coupled with an increased flux of the TCA cycle. They offer building blocks and energy for rapid cell proliferation [46]. Choline functions as an important methylation donor that can affect DNA methylation [47] and lead to disruption of DNA repair [48]. Further, choline, glycerophosphocholine, and lipids are essential for cell membrane synthesis and phospholipid metabolism, linking these metabolites to the cell cycle and increased cell turnover [49]. Elevated levels of phosphocholine and increased cell turnover is a well-known feature of prostate cancer [50].

In addition, the genes are strongly correlated (Spearman’s *ρ* > 0.5) with several CpGs (Supplementary Fig. 4). These are enriched in PRC and enhancer regions (Fig. 4C) which showed increased methylation in samples with cancer compared to those without cancer, and highest methylation in samples with aggressive disease. Further, the strongly correlated CpGs were also enriched in binding regions of several TFs (Fig. 4D), including MYC, CTCF, E2F6, CTCFL, and SP1, which are known to regulate proliferation of prostate cancer cells [51, 52].

We validated the cell cycle related genes as a gene signature and found a significant separation between the three risk groups (Fig. 4E; Supplementary Fig. 8).

The metabolites, genes, and methylation patterns associated with the cell cycle theme are not directly connected through a single pathway but are all essential for cell division. Gene expression regulates the mitotic spindle checkpoint, metabolism synthesizes cell membrane, and methylation rearranges chromatin to allow for cell cycle progression. Together, these processes promote cell proliferation.

### Smooth muscle-specific omics characteristics correlate with stromal enrichment

**Fig. 5:**
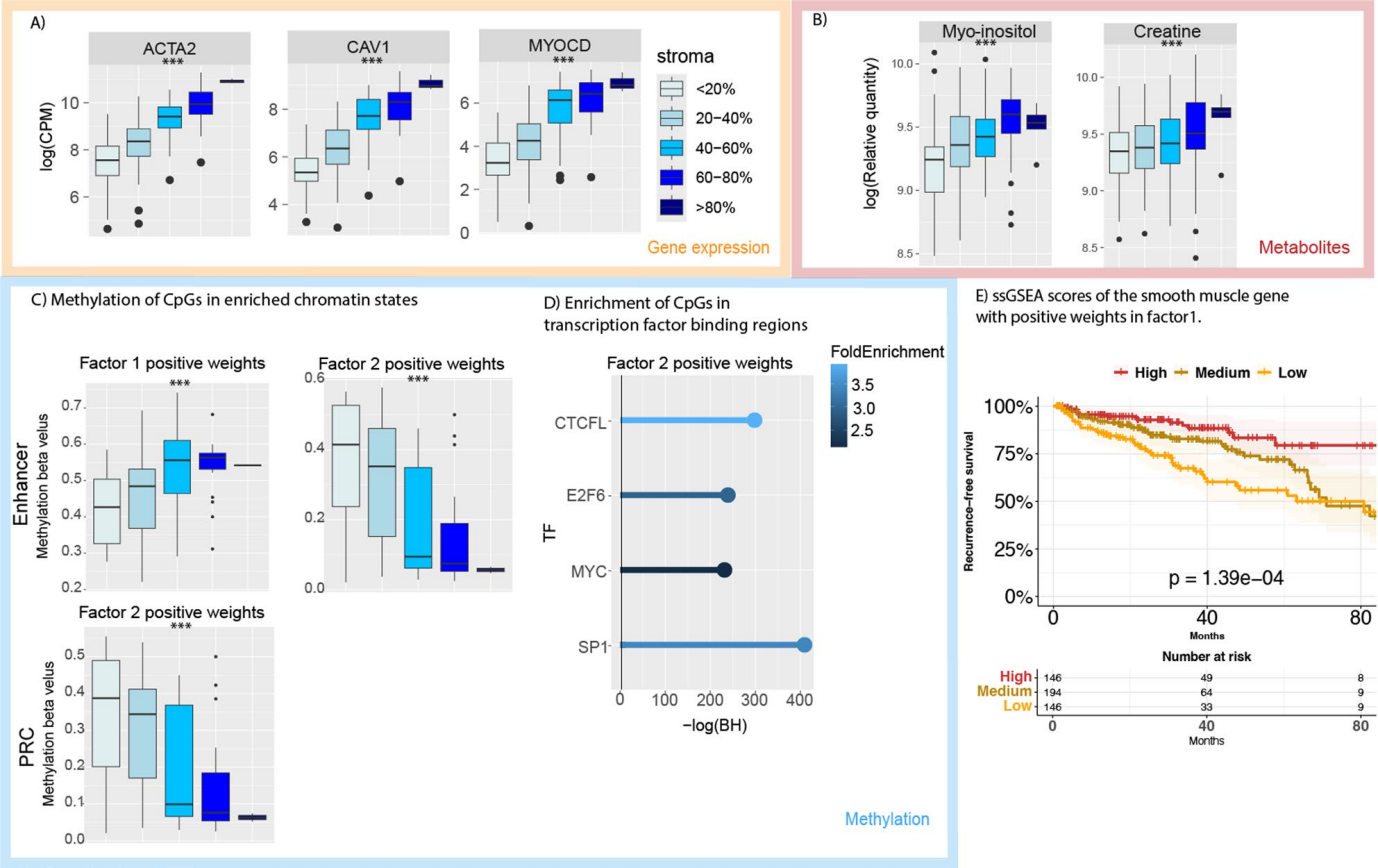
Smooth muscle. **A** Expression levels of positively weighted genes from Factors 1 and 2, including stromal and smooth muscle-associated genes (ACTA2, CAV1, and MYOCD) and immune related CD38 gene, across samples with varying stromal proportions. **B** Abundance of stromal-related metabolites (Myo-inositol and Creatine) in relation to tissue stromal content. Color code follows from A. **C** Methylation of CpGs in chromatin states enriched for enhancer and polycomb repressive complex (PRC). An inverse trend of enrichment with stromal content was observed between Factors 1 and 2. Color code follows from A. **D** Enrichment of CpGs in transcription factor binding regions, highlighting associations with CTCF, E2F6, MYC, and SP1 binding sites for positive weights in Factor 2. **E** Single-sample gene set enrichment analysis (ssGSEA) of smooth muscle-related genes in the TCGA prostate cancer cohort, showing lower recurrence-free survival among patients with low smooth muscle gene expression scores. * = *P*-value < 0.5; ** = *P*-value < 0.1; *** *P*-value < 0.01

The top positively weighted genes for the “smooth muscle” theme in Factors 1 and 2 included ACTA2 and MYOCD, which are canonical markers and regulators of smooth muscle differentiation, along with CAV1, a gene broadly expressed in stromal and vascular cells. Interestingly, expression levels of ACTA2, CAV1, and MYOCD increase progressively with higher stromal content. ACTA2 is primarily expressed in the smooth muscle cells of the prostate stroma, and downregulation of ACTA2 expression has been linked to poorer overall survival in prostate cancer patients [53]. Similarly, loss of CAV1 in the stroma is a strong marker for increased prostate cancer progression [54, 55]. Although MYOCD has not been explicitly studied in the context of prostate cancer, it is recognized as a tumor suppressor in lung cancer [56]. The observed higher expression of stromal genes in regions with abundant stroma suggests that Factors 1 and 2 may reflect normal prostate stromal characteristics. Moreover, the observed higher expression of smooth muscle genes mirrored the higher levels of metabolites such as myo-inositol and creatine in samples with a higher stroma content (Fig. 5A and B). We have previously detected creatine at higher levels in stroma with spatially resolved mass spectrometry imaging [32]. Creatine plays a vital role in muscle cells by regenerating ATP through the phosphocreatine–creatine kinase system and thereby maintaining ATP homeostasis during high-energy situations [57]. The exogenous myo-inositol functions as an EMT inhibitor [58], and increased levels in stroma-rich samples may suggest a local stromal defense.

Factor 1 and Factor 2 captured distinct CpG methylation patterns within enriched chromatin states, where Factor 1 showed an increase and Factor 2 a decrease in enhancer methylation beta values with increasing stromal content. This highlights two opposite stromal-associated epigenetic re-programming patterns (Fig. 5C). High-weight probes from both Factors 1 and 2 are provided in Supplementary Fig. 4. CpGs were enriched in transcription factor binding sites (including CTCFL, E2F6, MYC, and SP1), pointing to regulatory mechanisms such as chromatin remodeling, oncogenic transcriptional activation and stromal remodeling programs (Fig. 5D). Importantly, analysis of the TCGA cohort showed that reduced activity of a smooth muscle gene program correlated with significantly worse recurrence-free survival also in the TCGA cohort, suggesting that preservation of smooth muscle-like stromal characteristics may be associated with a more favorable prognosis (Fig. 5E; Supplementary Fig. 9).

### Different sets of genes are associated with immune activation in cancer and normal samples, and are related to DNA methylation changes (in enhancer and polycomb genomic regions)

GO terms related to immune activation are enriched in Factors 1, 2 and 4 (Fig. 2D), with Factors 1 and 4 stronger than Factor 2. In addition to RNA and metabolites, these three factors are characterized by being strongly influenced by changes in DNA methylation (Fig. 2C). There is a slight overall association with ISUP grade and delated clinical variables (clinical t-stage, seminal vesicle invasion, recurrence status, metastasis status and castrate resistance, Fig. 2B), but for sample characteristics there is no overall strong association with cancer and stroma content.

**Fig. 6:**
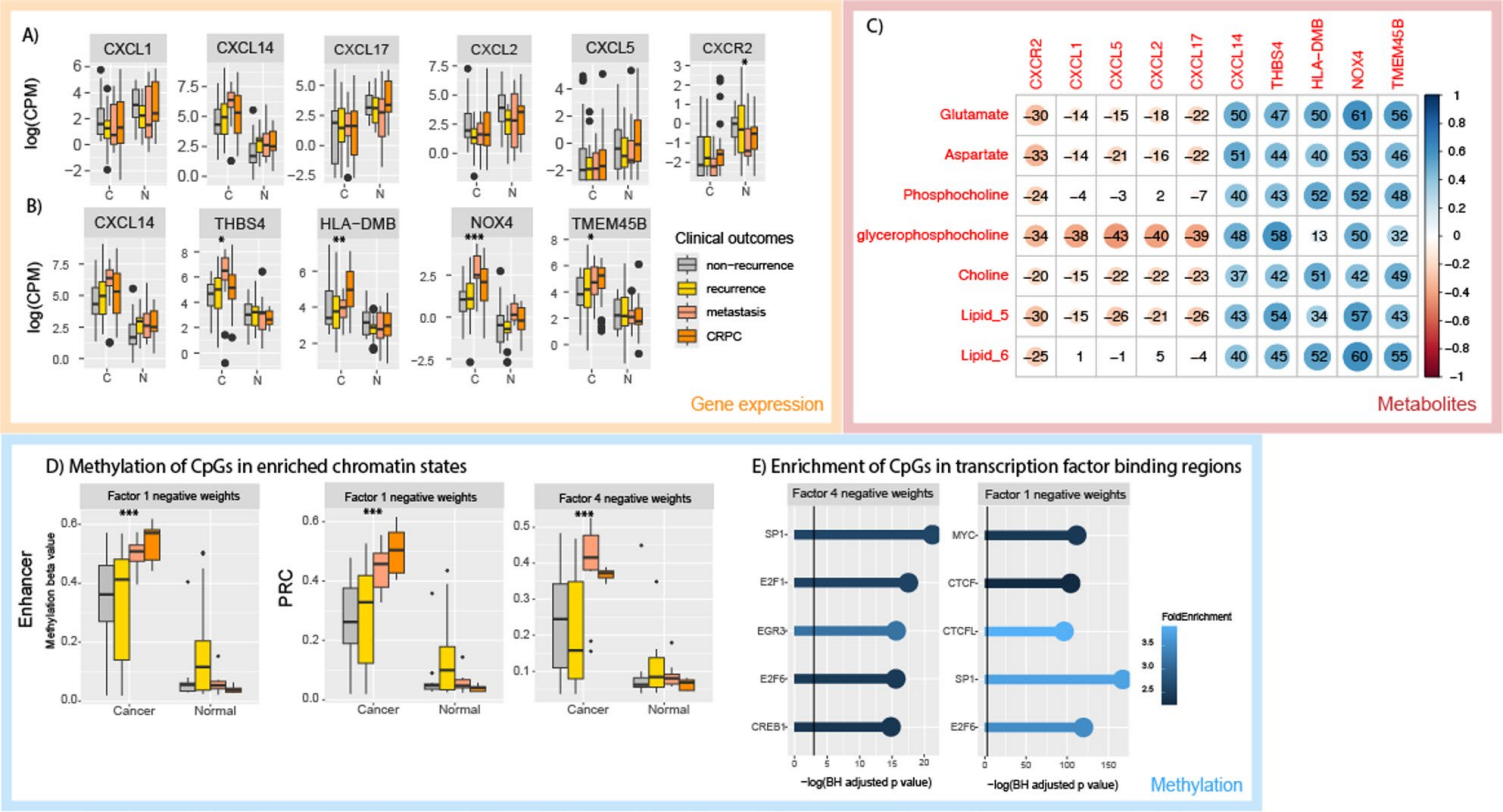
Immune activation. **A** Gene with strong weights with elevated expression in normal (N) samples is shown with their association with cancer aggressiveness. The immune activation genes upregulated in normal samples included several C-X-C motif chemokine ligands (CXCL). **B** Genes with strong weights with elevated expression in cancer (**C**) samples are shown with their association with cancer aggressiveness. **C** Correlation between genes with strong weights from (**A**) and (**B**) with metabolite levels. The immune-related genes associated with cancer samples are positively correlated with metabolic glutamate levels and changes to lipid metabolism citrate and polyamine levels. **D** Methylation sites with strong weights in Factors 1 and 4 and their overlap with chromatin states characterizing enhancers and polycomb repression (PRC), suggesting that epigenetic changes can be a driver for immune related changes in cancer development. **E** Methylation sites with strong weights in Factors 1, 2 and 4 are enriched for binding sites for transcription factors involved in chromatin remodeling and proliferation. * = *P*-value < 0.5; ** = *P*-value < 0.1; *** *P*-value < 0.01

We identified two subsets of immune-related genes associated with the three immune activation factors, where one subset was more enriched in cancer samples and one subset was more enriched in normal samples. The subset enriched in normal samples included several C-X-C motif chemokine ligands (CXCL) (Fig. 6A). Interestingly, three of these chemokines (CXCL17, CXCL2 and CXCL5) are part of a gene signature recently published by our research group which characterize local immune modulation and invasion of club-cells in normal appearing glands adjacent to aggressive tumors [59]. This demonstrates that the *MOFA-*factorization can capture trends in local tissue compartments which otherwise could only be identified by more advanced methods such as Spatial Transcriptomics. The immune-related genes that MOFA associated with cancer samples were positively correlated with glutamate levels and changes in lipid levels (Fig. 6C).

We observed a strong association of Factors 1, 2 and 4 with changes in DNA Methylation. Methylation site changes with high absolute weights in these factors are enriched in enhancers and PRC regulatory regions, and these changes are more prominent in aggressive cancer samples (Fig. 6D). This suggests that the epigenetic changes can be driven by immune-related processes in cancer development. These methylation sites were also enriched for transcription factors known to be involved in chromatin remodeling (CTCF and CTCFL) [38, 39] and tumor proliferation (MYC, E2F, SP1) (Fig. 6E) [52, 60–62]. These changes can be caused both by active chromatin remodeling of prostate epithelial cells or by infiltration of foreign cell-types (for example Club cells) in the tissue environment. However, changes in polycomb-regulation are generally associated with developmental programs [63].

Although the Kaplan-Meier analysis showed no significant outcome for patients with intermediate levels of immune related genes (Supplementary Fig. 10–12), our immune activation results may indicate that balanced immune activation is more beneficial for tumor development [64].

### Stromal and glandular morphology gene features show an inverse association to cancer characteristics

GO terms related to morphology and tissue development (*n* = 8) all showed a similar pattern across Factors 3, 4 and 5 with negative feature weights (Fig. 2D). Tissue morphology refers to the structural organization of tissue. In prostate, the key tissue compartments are the glandular structures and the surrounding stroma. Factor 3 correlated strongly with both stroma content and cancer content, though in opposite directions (Fig. 2B), whereas these relationships were much weaker for Factors 4 and 5.

**Fig. 7:**
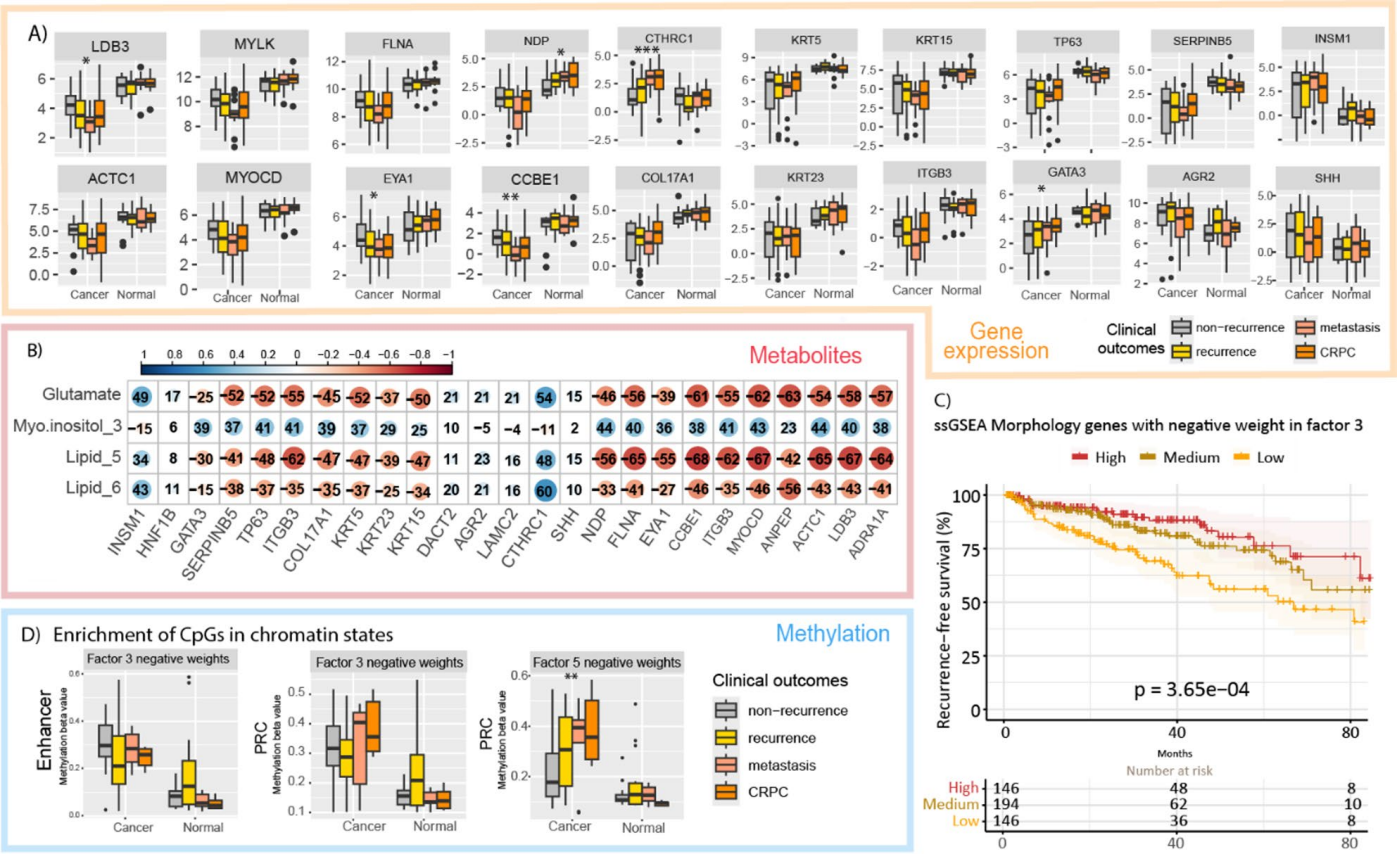
Tissue morphology. **A** Gene expression of morphology genes related to stroma and glandular morphology with negative feature weights in Factors 3, 4, and 5. **B** Correlation plot (Spearman) between morphology genes and metabolites. **C** Kaplan-Meier survival analysis based on GSEA with the morphology genes with negative feature weights for Factor 3. **D** Enrichment of CpGs in chromatin states across negative Factors 3 and 5. * = P-value < 0.5; ** = P-value < 0.1; *** = P-value < 0.01

Four of the eight enriched GO terms were related to gland morphology and one to appendage development, while three GO-terms gave the indication of enriched vasculature function. However, further inspection of the genes within the vasculature GO-terms and interpreting them in the context of prostate tissue biology, indicated that this likely represents enrichment of stroma-related gene expression, rather than vasculature morphology. This is showcased by enrichment of MYLK, MYOCD, ACTC1, LDB3 and FLNA which are all key genes of muscle fibers of smooth muscle cells [65]. These genes had the strongest negative factor weights for Factor 3 (compared to Factors 4 and 5) and systematically had the highest expression in normal prostate samples (Fig. 7a). Further, all these stroma genes had a high inverse correlation (Spearman’s *ρ* => -0.55) with Lipid_5 (Fig. 7b). As previously presented, Lipid_5 showed higher levels in cancer samples compared to normal samples (Fig. 4B). Although we do not have the precise identity of the lipids contributing to this NMR-signal (ppm-range 3.68–3.79), higher lipid concentration is a well-known characteristic of prostate cancer [50]. It is therefore expected that stroma-genes associated with healthy tissue have an inverse relationship with lipids.

Exploring the genes within gland-morphology GO terms showed that most were linked to basal cells, which help maintain normal prostate gland integrity. Proteins that contribute to this physical integrity make up the intermediate filaments and include cytokeratin genes such as KRT5, KRT15 and KRT23 which had strong negative feature weights for both Factors 3 and 5 and were downregulated in cancer samples (Fig. 7a). As loss of basal cells is one of the first indications of cancer, cytokeratin 5 (KRT5) is routinely used in the clinic to confirm the presence of basal cells and to differentiate benign glands from invasive carcinoma [66]. Another clinically used marker for basal cells that had high negative feature weights (Factors 3 and 5) is TP63 (Fig. 7A). TP63 (p63) is a transcription factor and a known tumor suppressor of prostate cancer [67]. In line with previous scientific consensus, TP63 had reduced expression in cancer samples (Fig. 7A) [67]. GATA3 is also a transcription factor with tumor suppressive properties that showed the same pattern [68]. Other genes related to basal cells and basement membrane with negative feature weights in Factors 3 and 5 included SERPINB5, ITGB3 and COL17A. TP63, ITGB3 and SERPINB5 had a strong inverse correlation with glutamate (Spearman’s ρ ≥ -0.52, Fig. 7B). As previously mentioned, glutamate was found at higher levels in cancer compared to normal tissue both in this and other studies [46]. Similarly to the stroma-related genes, the gland genes were also inversely correlated with Lipid_5.

Negative Factor 3 is capturing features related to normal prostate tissue morphology, indicated by genes known to be present in healthy stroma and glands and by the stroma content pulling in the same direction (Fig. 2B). On the other hand, cancer content and recurrence status had an inverse relationship with negative Factor 3 (Fig. 2B). This inverse association to clinical outcome was validated in the TCGA-cohort, where a high ssGSEA score for morphology-genes (with high negative feature weights in Factor 3) was protective against recurrence (*p* = 0.0004, Fig. 7C, Supplementary Figs. 13–14).

Some genes within this morphology theme only had negative feature weights for Factor 5 and generally were less prominent in Factors 3 and 4 (Supplementary Table 1). Interestingly, several of these genes, including INSM1, SHH and AGR2 had an increased expression in cancer samples compared to normal (Fig. 7a). These genes have more diverse functions with INSM1 being a transcription factor [69], while AGR2 is involved in protein folding [70] and SHH is important prostate development and regeneration [71]. Factor 5 is clearly capturing other morphology functions that are not highly associated with neither normal or cancer morphology but rather represents variation that is independent of stroma and cancer content.

The identified CpGs with negative weights in Factors 3 and 5 were enriched in enhancers and PRC regions (Supplementary Fig. 4) and were associated with clinical outcomes (Fig. 7D). The CpG enrichments in enhancers and PRC regions were in line with the other factors and biological themes, suggesting that chromatin remodeling plays a part in several cancer driving biological functions of prostate cancer.

## Discussion and concluding remarks

Our multi-omics analysis of both normal and cancerous prostate tissues has unveiled several distinct biological themes associated with prostate cancer and prostate cancer aggressiveness. The advantage of employing MOFA in this study lies in its ability to integrate heterogeneous data modalities while robustly handling missing observations across datasets. Although this linear framework may not capture complex non-linear relationships, its high interpretability proved valuable for elucidating the connections between molecular features and prostate cancer biology. Consistent with the model’s architecture, Factor 1 accounted for the greatest proportion of variance across all three omics layers and exhibited the strongest correlations with clinical characteristics (Fig. 2A, B). This suggests that the dominant source of variation in the dataset is inherently linked to cancer biology and disease progression. Furthermore, the highest enriched GO terms found in Factor 1 were related to cell cycle (Fig. 2D). Dysregulation of the cell cycle is a particularly well-known hallmark of cancer [44, 45], and these findings therefore prove that MOFA is accurately capturing the important variation associated with cancer aggression. To further validate the biological relevance of our results, we performed a survival analysis on the TCGA dataset using gene signatures derived from biological themes. We used the ssGSEA method since it is unsupervised and therefore a suitable and robust approach for identifying whether a biological difference between recurrent and non-recurrent patients exists without training.

The precision of MOFA allowed us to link aspects of prostate cancer molecular biology that had not been previously studied across multiple omics levels. The expression of metallothioneins and their potential connection to citrate have been documented [33], but our study suggest that they might be regulated by promoter methylation. Similarly, the mitotic spindle checkpoint in prostate cancer has traditionally been examined one gene at a time, but we identified a pattern of increased expression across multiple checkpoint genes. Although cancer-related metabolism and the loss of basal cell fate are well-known phenomena [72, 73], our study suggest they might be regulated by chromatin state rearrangements. While the immune microenvironment in prostate cancer has been described in single-cell or spatial contexts [74–77], our study pinpoints chemokines and immunoglobulins as important elements of the prostate cancer tumor microenvironment.

Factors 1 and 3 captured molecular variations associated with ISUP grade, cancer content, and stroma content, which are challenging to disentangle using bulk data. Prostate cancer originates from epithelial cells and will therefore be a source of epithelial genes and regulatory mechanisms. Stroma, on the other hand, is displaced by growing tumors, and thus a “normal” sample also includes more stromal tissue than one with cancer, leading to imbalanced stroma signals.

In addition, samples with higher ISUP grade often have a larger cancer area and thus less stroma content introducing a bias in the analyses. Due to this, separating biological mechanisms coming from ISUP grade, cancer content and stroma is not feasible with the current experimental design. A clear next step is to explore the biological patterns from this study further using spatial technologies and/or dissected tissue compartments.

Our integrative MOFA analysis revealed that CpGs contributing to multiple molecular factors in prostate cancer are enriched both in PRC-associated chromatin states (Figs. 3, 4, 5, 6 and 7) and in binding regions of the transcription factors SP1 and CTCFL (BORIS). This enrichment points to an epigenetic landscape in which developmental regulatory elements, normally repressed by Polycomb, become accessible and engaged by transcriptional activators [78]. CTCFL is known to compete for binding sites with CTCF [79] and may potentially displace PRC2 and facilitate chromatin opening [80]. SP1 can occupy CpG-rich promoters upon chromatin opening to initiate transcriptional activation [81]. Together, these mechanisms suggest a coordinated reactivation of Polycomb-silenced gene programs during prostate tumorigenesis.

In our analyses, SP1 and CTCFL emerge as recurrent transcriptional regulators shaping diverse biological processes captured by the MOFA factors. SP1 is a well-characterized factor in prostate cancer, promoting androgen receptor transcriptional activity [82], and its activity is often enhanced by promoter hypomethylation and increased chromatin accessibility [83]. In contrast, CTCFL, a testis-specific paralog of CTCF, is only sporadically reported in prostate cancer but has been implicated in DNA hypomethylation and chromatin reorganization in other malignancies [84, 85]. Its ectopic activation may alter enhancer insulation and enable germline-like gene expression. The consistent co-enrichment of SP1 and CTCFL binding regions among factor-associated CpGs across functional modules such as cell cycle, zinc metabolism, immune signaling and stromal remodeling supports a model of global epigenetic reprogramming. These findings together point to a novel interplay between Polycomb deregulation, CTCFL-mediated chromatin remodeling, and SP1-driven transcriptional activation in shaping the prostate cancer epigenome.

A limitation of this study is the unbalanced representation across omics layers (Fig. 2A), as data for all modalities could not be obtained for every sample. This was due to multifactorial constraints, including limited time, resources, and the high analytical costs associated with DNA methylation and gene expression profiling. MOFA is designed to handle such missing measurements, however, extra care must be taken when interpreting the cumulative variance explained in the MOFA model. As noted by the MOFA developers, having fewer samples present in one omics dataset tend to result in more variance captured from that omics dataset [86]. This is likely the case in our MOFA model, where the DNA methylation dataset (*n* = 96) had the highest cumulative variance explained of 76% compared to the transcriptomics (50%, *n* = 176) and metabolomics data (29%, *n* = 280). Furthermore, the metabolic coverage was restricted to 31 metabolites via HRMAS analyses. However, the use of this non-destructive technique was essential to enable subsequent gene expression analysis on the same physical samples. Notably, the identified metabolites represent key oncogenic pathways, including the TCA cycle, amino acid metabolism, and lipid levels. While MOFA produced robust factors that model the linear relationship between different omics layers, it is possible that non-linear relationships were not captured. A follow-up study could focus more strongly on identifying such more subtle patterns.

To conclude, by integrating DNA methylation, gene expression and metabolites, we show that key prostate cancer-driving biological functions are impacted by multiple molecular alterations. Metallothionein/citrate levels, cell cycle activity, smooth muscle/stromal integrity, immune activation and tissue morphology are all functions affected non-redundantly by all three molecular layers, supporting a system-level deregulation of these key functions. The alterations in CpGs in PRC regions and binding regions of SP1 and CTCFL suggest that epigenetic reprogramming contributes to these coordinated transcriptional and metabolomic changes in prostate cancer. Gene expression signatures derived from these biological functions show validated prognostic power illustrating both biological and clinical relevance.

## Supplementary Information

Supplementary Material 1.

Supplementary Material 2.

## Data Availability

The data generated and analyzed in this study includes sensitive information, and its management must comply with the General Data Protection Regulation (GDPR), Norwegian law, and the specific patient consent and ethical approval. Consequently, the data is legally subjected to restricted access. Raw and processed transcriptomics and DNA methylation data have been deposited at Federated European Genome-Phenome Archive (FEGA) Norway and are findable on the EGA portal (ega-archive.org) under the study ID EGAS50000000413. The transcriptomics and DNA methylation data are deposited as separate datasets with the accession numbers EGAD50000000604 and EGAD50000000605, respectively. Data access can be requested through the EGA portal, where any data request will be processed through a data access committee at NTNU. HR-MAS NMR, stained images, and tissue annotations are not externally archived as there are currently no suitable public data repositories that accept such sensitive data and that meet the data sharing criteria postulated by the study’s ethical approval, patient consent, GDPR and Norwegian law. Access to all the above data will only be granted after the following steps have been achieved; (1) the data requester and the intended use of the data must comply with GDPR regulation, Norwegian law, and the specific patient consent, (2) data sharing with the specific data requester must be approved by the regional ethical committee (REC) in Norway, (3) the Data Protection Impact Assessment (DPIA) may require revision and (4) there must be a signed data transfer agreement between the institution of the data requester and NTNU. Depending on the intended use of the data, the data requester can also be required to establish a collaboration agreement with NTNU prior to data sharing. ChromHMM segmentation data were downloaded from the ENCODE data portal from two prostate cancer cell lines: PC-3 (Accession ENCSR103ZFL) and LNCaP (Accession ENCSR072ZGM). Maps of direct transcription factor-DNA (TF-DNA) interactions were obtained from the UniBind database (https://unibind.uio.no). Bulk RNA-seq expression data for validation of gene signatures were obtained from cbioportal database (https://www.cbioportal.org/datasets) under the heading Prostate Adenocarcinoma (TCGA, PanCancer Atlas). Clinical metadata for TCGA-PRAD were acquired from portal.gdc.cancer.gov/projects/TCGA-PRAD.

## Abbreviations

BCR: Biochemical recurrence
BORIS: Binding regions of the transcription factors SP1 and CTCFL
CPM: Counts per million
CRPC: Castrate resistance
CXCL: C-X-C motif chemokine ligands
DPIA: Data Protection Impact Assessment
EMT: Epithelial-mesenchymal transition
FDR: False discovery rate
FEGA: Federated European Genome-Phenome Archive
GDPR: General Data Protection Regulation
GO: Gene ontology
GSEA: Gene set enrichment analysis
HES: Hematoxylin, erythrosine, and saffron
HRMAS: High-resolution magic angle spinning
IQR: Inter-quartile range
ISUP: International Society for Urological Pathology
MOFA: Multi-omics factor analysis framework
NMR: Nuclear magnetic resonance
PSA: Prostate-specific antigen
PWM: Position weight matrix
PRC: Polycomb repressive complex
REC: Regional ethical committee
RP: Radical prostatectomy
ssGSEA: Single-sample gene set enrichment analysis
TFBS: Transcription factor binding site
TFBR: Transcription factor binding regions
TF-DNA: Transcription factor – DNA

## References

[1] Lloyd T, Hounsome L, Mehay A, Mee S, Verne J, Cooper A. Lifetime risk of being diagnosed with, or dying from, prostate cancer by major ethnic group in England 2008–2010. BMC Med. 2015;13:171. 26224061 10.1186/s12916-015-0405-5 PMC4520076

[2] Gandaglia G, Leni R, Bray F, Fleshner N, Freedland SJ, Kibel A, et al. Epidemiology and Prevention of Prostate Cancer. Eur Urol Oncol. 2021;4(6):877–92. 34716119 10.1016/j.euo.2021.09.006

[3] Figiel S, Yin W, Doultsinos D, Erickson A, Poulose N, Singh R, et al. Spatial transcriptomic analysis of virtual prostate biopsy reveals confounding effect of tissue heterogeneity on genomic signatures. Mol Cancer. 2023;22(1):162. 37789377 10.1186/s12943-023-01863-2 PMC10546768

[4] Tolkach Y, Kristiansen G. The Heterogeneity of Prostate Cancer: A Practical Approach. Pathobiology. 2018;85(1–2):108–16. 29393241 10.1159/000477852

[5] Subramanian I, Verma S, Kumar S, Jere A, Anamika K. Multi-omics Data Integration, Interpretation, and Its Application. Bioinform Biol Insights. 2020;14:1177932219899051. 32076369 10.1177/1177932219899051 PMC7003173

[6] Argelaguet R, Arnol D, Bredikhin D, Deloro Y, Velten B, Marioni JC, et al. MOFA+: a statistical framework for comprehensive integration of multi-modal single-cell data. Genome Biol. 2020;21(1):111. 32393329 10.1186/s13059-020-02015-1 PMC7212577

[7] Steffen PA, Ringrose L. What are memories made of? How Polycomb and Trithorax proteins mediate epigenetic memory. Nat Rev Mol Cell Biol. 2014;15:340. 24755934 10.1038/nrm3789

[8] Varambally S, Dhanasekaran SM, Zhou M, Barrette TR, Kumar-Sinha C, Sanda MG, et al. The polycomb group protein EZH2 is involved in progression of prostate cancer. Nature. 2002;419(6907):624–9. 12374981 10.1038/nature01075

[9] Massie CE, Mills IG, Lynch AG. The importance of DNA methylation in prostate cancer development. J Steroid Biochem Mol Biol. 2017;166:1–15. 27117390 10.1016/j.jsbmb.2016.04.009

[10] Bertilsson H, Angelsen A, Viset T, Skogseth H, Tessem M-B, Halgunset J. A new method to provide a fresh frozen prostate slice suitable for gene expression study and MR spectroscopy. Prostate. 2011;71(5):461–9. 20860008 10.1002/pros.21260

[11] Wang T, Shao K, Chu Q, Ren Y, Mu Y, Qu L, et al. Automics: an integrated platform for NMR-based metabonomics spectral processing and data analysis. BMC Bioinformatics. 2009;10:83. 19291281 10.1186/1471-2105-10-83 PMC2666662

[12] Eilers P, Boelens H. Baseline Correction with Asymmetric Least Squares Smoothing. Unpubl Manuscr. 2005.

[13] Savorani F, Tomasi G, Engelsen SB. icoshift: A versatile tool for the rapid alignment of 1D NMR spectra. J Magn Reson. 2010;202(2):190–202. 20004603 10.1016/j.jmr.2009.11.012

[14] Giskeødegård GF, Bertilsson H, Selnæs KM, Wright AJ, Bathen TF, Viset T, et al. Spermine and Citrate as Metabolic Biomarkers for Assessing Prostate Cancer Aggressiveness. PLoS ONE. 2013;8(4):e62375. 23626811 10.1371/journal.pone.0062375 PMC3633894

[15] Hansen AF, Sandsmark E, Rye MB, Wright AJ, Bertilsson H, Richardsen E, et al. Presence of TMPRSS2-ERG is associated with alterations of the metabolic profile in human prostate cancer. Oncotarget. 2016;7(27):42071–85. 27276682 10.18632/oncotarget.9817 PMC5173117

[16] Braadland PR, Giskeødegård G, Sandsmark E, Bertilsson H, Euceda LR, Hansen AF, et al. Ex vivo metabolic fingerprinting identifies biomarkers predictive of prostate cancer recurrence following radical prostatectomy. Br J Cancer. 2017;117(11):1656–64. 28972967 10.1038/bjc.2017.346 PMC5729443

[17] Liao Y, Smyth GK, Shi W. featureCounts: an efficient general purpose program for assigning sequence reads to genomic features. Bioinformatics. 2013;30(7):923–30. 24227677 10.1093/bioinformatics/btt656

[18] Zhang Y, Parmigiani G, Johnson WE. ComBat-seq: batch effect adjustment for RNA-seq count data. NAR Genomics Bioinf. 2020;2(3). 10.1093/nargab/lqaa078 PMC7518324 33015620

[19] Zhou W, Laird PW, Shen H. Comprehensive characterization, annotation and innovative use of Infinium DNA methylation BeadChip probes. Nucleic Acids Res. 2017;45(4):e22. 27924034 10.1093/nar/gkw967 PMC5389466

[20] Aryee MJ, Jaffe AE, Corrada-Bravo H, Ladd-Acosta C, Feinberg AP, Hansen KD, et al. Minfi: a flexible and comprehensive Bioconductor package for the analysis of Infinium DNA methylation microarrays. Bioinformatics. 2014;30(10):1363–9. 24478339 10.1093/bioinformatics/btu049 PMC4016708

[21] Johnson WE, Li C, Rabinovic A. Adjusting batch effects in microarray expression data using empirical Bayes methods. Biostatistics. 2007;8(1):118–27. 16632515 10.1093/biostatistics/kxj037

[22] Du P, Zhang X, Huang CC, Jafari N, Kibbe WA, Hou L, et al. Comparison of Beta-value and M-value methods for quantifying methylation levels by microarray analysis. BMC Bioinformatics. 2010;11:587. 21118553 10.1186/1471-2105-11-587 PMC3012676

[23] Xi Y, Shi J, Li W, Tanaka K, Allton KL, Richardson D, et al. Histone modification profiling in breast cancer cell lines highlights commonalities and differences among subtypes. BMC Genomics. 2018;19(1):150. 29458327 10.1186/s12864-018-4533-0 PMC5819162

[24] Luo Y, Hitz BC, Gabdank I, Hilton JA, Kagda MS, Lam B, et al. New developments on the Encyclopedia of DNA Elements (ENCODE) data portal. Nucleic Acids Res. 2020;48(D1):D882–9. 31713622 10.1093/nar/gkz1062 PMC7061942

[25] Quinlan AR, Hall IM. BEDTools: a flexible suite of utilities for comparing genomic features. Bioinformatics. 2010;26(6):841–2. 20110278 10.1093/bioinformatics/btq033 PMC2832824

[26] Gheorghe M, Sandve GK, Khan A, Chèneby J, Ballester B, Mathelier A. A map of direct TF-DNA interactions in the human genome. Nucleic Acids Res. 2019;47(4):e21. 30517703 10.1093/nar/gky1210 PMC6393237

[27] Khan A, Fornes O, Stigliani A, Gheorghe M, Castro-Mondragon JA, van der Lee R, et al. JASPAR 2018: update of the open-access database of transcription factor binding profiles and its web framework. Nucleic Acids Res. 2018;46(D1):D260–6. 29140473 10.1093/nar/gkx1126 PMC5753243

[28] Hänzelmann S, Castelo R, Guinney J. GSVA: gene set variation analysis for microarray and RNA-Seq data. BMC Bioinformatics. 2013;14(1):7. 23323831 10.1186/1471-2105-14-7 PMC3618321

[29] Biecek AKaMKaP. survminer: Drawing Survival Curves using ‘ggplot2’. 2025.

[30] Takahashi S. Positive and negative regulators of the metallothionein gene (review). Mol Med Rep. 2015;12(1):795–9. 25760317 10.3892/mmr.2015.3459

[31] Costello LC, Franklin RB. A comprehensive review of the role of zinc in normal prostate function and metabolism; and its implications in prostate cancer. Arch Biochem Biophys. 2016;611:100–12. 27132038 10.1016/j.abb.2016.04.014 PMC5083243

[32] Andersen MK, Høiem TS, Claes BSR, Balluff B, Martin-Lorenzo M, Richardsen E, et al. Spatial differentiation of metabolism in prostate cancer tissue by MALDI-TOF MSI. Cancer Metabolism. 2021;9(1):9. 33514438 10.1186/s40170-021-00242-z PMC7847144

[33] Rye MB, Krossa S, Hall M, van Mourik C, Bathen TF, Drabløs F, et al. The genes controlling normal function of citrate and spermine secretion are lost in aggressive prostate cancer and prostate model systems. iScience. 2022;25(6):104451. 35707723 10.1016/j.isci.2022.104451 PMC9189124

[34] Deaton AM, Bird A. CpG islands and the regulation of transcription. Genes Dev. 2011;25(10):1010–22. 21576262 10.1101/gad.2037511 PMC3093116

[35] Huang H, Zhang W. The SP1/SNHG16/GLUT1 axis promotes prostate cancer proliferation and invasion by regulating glucose metabolism. Archives Med Sci. 2025;21(2):638–47. 10.5114/aoms/191153 PMC12087323 40395899

[36] Yang M, Li LJ, Qiu GP, Liu H, Gao F. Specificity protein 1 initiates epithelial-mesenchymal transition of circulating tumor cells to inhibit metastasis in prostate cancer. Cancer Cell Int. 2025;25(1):255. 40646523 10.1186/s12935-025-03888-7 PMC12247201

[37] Peng S, Lin W, Li Z, Zhuang R, Peng S, Li B, et al. Cancer cell-intrinsic cholesterol induces lipid-associated macrophage differentiation via SP1 palmitoylation to promote prostate cancer progression. Adv Sci (Weinh). 2026;13(19):e08588. 10.1002/advs.202508588 PMC13045348 41603134

[38] CheemaZ, Hari‐Gupta Y, Kita GX, Farrar D, Seddon I, Corr J, et al. Expression of the cancer‐testis antigen BORIS correlates with prostate cancer. The Prostate. 2014;74(2):164–76. 10.1002/pros.22738. 10.1002/pros.22738 24123052

[39] Klenova EM, Morse HC 3rd, Ohlsson R, Lobanenkov VV. The novel BORIS + CTCF gene family is uniquely involved in the epigenetics of normal biology and cancer. Semin Cancer Biol. 2002;12(5):399–414. 10.1016/s1044-579x(02)00060-3 12191639

[40] Boormans JL, Korsten H, Ziel-van der Made AJC, van Leenders GJLH, de Vos CV, Jenster G, et al. Identification of TDRD1 as a direct target gene of ERG in primary prostate cancer. Int J Cancer. 2013;133(2):335–45. 23319146 10.1002/ijc.28025

[41] Chen S, Wang J, Wang L, Peng H, Xiao L, Li C, et al. Silencing TTK expression inhibits the proliferation and progression of prostate cancer. Exp Cell Res. 2019;385(1):111669. 31605696 10.1016/j.yexcr.2019.111669

[42] Weaver BAA, Cleveland DW. Decoding the links between mitosis, cancer, and chemotherapy: The mitotic checkpoint, adaptation, and cell death. Cancer Cell. 2005;8(1):7–12. 16023594 10.1016/j.ccr.2005.06.011

[43] Kops GJPL, Weaver BAA, Cleveland DW. On the road to cancer: aneuploidy and the mitotic checkpoint. Nat Rev Cancer. 2005;5(10):773–85. 16195750 10.1038/nrc1714

[44] Stopsack KH, Whittaker CA, Gerke TA, Loda M, Kantoff PW, Mucci LA et al. Aneuploidy drives lethal progression in prostate cancer. Proceedings of the National Academy of Sciences. 2019;116(23):11390-5. 10.1073/pnas.1902645116 PMC6561291 31085648

[45] Carceles-Cordon M, Orme JJ, Domingo-Domenech J, Rodriguez-Bravo V. The yin and yang of chromosomal instability in prostate cancer. Nat Reviews Urol. 2024;21(6):357–72. 10.1038/s41585-023-00845-9 PMC11156566 38307951

[46] Jain M, Nilsson R, Sharma S, Madhusudhan N, Kitami T, Souza AL, et al. Metabolite Profiling Identifies a Key Role for Glycine in Rapid Cancer Cell Proliferation. Science. 2012;336(6084):1040–4. 22628656 10.1126/science.1218595 PMC3526189

[47] Wen S, He Y, Wang L, Zhang J, Quan C, Niu Y, et al. Aberrant activation of super enhancer and choline metabolism drive antiandrogen therapy resistance in prostate cancer. Oncogene. 2020;39(42):6556–71. 32917955 10.1038/s41388-020-01456-z

[48] Awwad HM, Geisel J, Obeid R. The role of choline in prostate cancer. Clin Biochem. 2012;45(18):1548–53. 22921309 10.1016/j.clinbiochem.2012.08.012

[49] Sonkar K, Ayyappan V, Tressler CM, Adelaja O, Cai R, Cheng M, et al. Focus on the glycerophosphocholine pathway in choline phospholipid metabolism of cancer. NMR Biomed. 2019;32(10):e4112. 31184789 10.1002/nbm.4112 PMC6803034

[50] Keshari KR, Tsachres H, Iman R, Delos Santos L, Tabatabai ZL, Shinohara K, et al. Correlation of phospholipid metabolites with prostate cancer pathologic grade, proliferative status and surgical stage - impact of tissue environment. NMR Biomed. 2011;24(6):691–9. 21793074 10.1002/nbm.1738 PMC3653775

[51] Silva KCS, Tambwe N, Mahfouz DH, Wium M, Cacciatore S, Paccez JD, et al. Transcription Factors in Prostate Cancer: Insights for Disease Development and Diagnostic and Therapeutic Approaches. Genes. 2024;15(4):450. 38674385 10.3390/genes15040450 PMC11050257

[52] Wei Z, Wang S, Xu Y, Wang W, Soares F, Ahmed M, et al. MYC reshapes CTCF-mediated chromatin architecture in prostate cancer. Nat Commun. 2023;14(1):1787. 36997534 10.1038/s41467-023-37544-3 PMC10063626

[53] Zhu H, Lin Q, Gao X, Huang X. Identification of the hub genes associated with prostate cancer tumorigenesis. Front Oncol. 2023;13:1168772. 10.3389/fonc.2023.1168772 PMC10213256 37251946

[54] Ayala G, Morello M, Frolov A, You S, Li R, Rosati F, et al. Loss of caveolin-1 in prostate cancer stroma correlates with reduced relapse-free survival and is functionally relevant to tumour progression. J Pathol. 2013;231(1):77–87. 23729330 10.1002/path.4217 PMC3978784

[55] Hammarsten P, Dahl Scherdin T, Hägglöf C, Andersson P, Wikström P, Stattin P, et al. High caveolin-1 expression in tumor stroma is associated with a favourable outcome in prostate cancer patients managed by watchful waiting. PLoS ONE. 2016;11(10):e0164016. 27764093 10.1371/journal.pone.0164016 PMC5072718

[56] Zhou Q, Chen W, Fan Z, Chen Z, Liang J, Zeng G, et al. Targeting hyperactive TGFBR2 for treating MYOCD deficient lung cancer. Theranostics. 2021;11(13):6592–606. 33995678 10.7150/thno.59816 PMC8120205

[57] Hargreaves M, Spriet LL. Skeletal muscle energy metabolism during exercise. Nat Metabolism. 2020;2(9):817–28. 10.1038/s42255-020-0251-4 32747792

[58] Bizzarri M, Dinicola S, Bevilacqua A, Cucina A. Broad Spectrum Anticancer Activity of Myo-Inositol and Inositol Hexakisphosphate. Int J Endocrinol. 2016;2016(1):5616807. 27795708 10.1155/2016/5616807 PMC5067332

[59] Krossa S, Andersen MK, Sandholm EM, Wess M, Kiviaho A, Sharma A, et al. Spatial multi-omics identifies aggressive prostate cancer signatures highlighting pro-inflammatory chemokine activity in the tumor microenvironment. Nat Commun. 2025;16(1):10160. 41258098 10.1038/s41467-025-65161-9 PMC12630738

[60] Kent LN, Leone G. The broken cycle: E2F dysfunction in cancer. Nat Rev Cancer. 2019;19(6):326–38. 31053804 10.1038/s41568-019-0143-7

[61] Johnson DG, Schneider-Broussard R. Role of E2F in cell cycle control and cancer. Front Biosci. 1998;3:d447–8. 9556498 10.2741/a291

[62] Safe S, Abbruzzese J, Abdelrahim M, Hedrick E. Specificity Protein Transcription Factors and Cancer: Opportunities for Drug Development. Cancer Prev Res (Phila). 2018;11(7):371–82. 29545399 10.1158/1940-6207.CAPR-17-0407

[63] Lee TI, Jenner RG, Boyer LA, Guenther MG, Levine SS, Kumar RM, et al. Control of developmental regulators by Polycomb in human embryonic stem cells. Cell. 2006;125(2):301–13. 16630818 10.1016/j.cell.2006.02.043 PMC3773330

[64] Tekpli X, Lien T, Røssevold AH, Nebdal D, Borgen E, Ohnstad HO, et al. An independent poor-prognosis subtype of breast cancer defined by a distinct tumor immune microenvironment. Nat Commun. 2019;10(1):5499. 31796750 10.1038/s41467-019-13329-5 PMC6890706

[65] Joseph DB, Henry GH, Malewska A, Reese JC, Mauck RJ, Gahan JC, et al. Single-cell analysis of mouse and human prostate reveals novel fibroblasts with specialized distribution and microenvironment interactions. J Pathol. 2021;255(2):141–54. 34173975 10.1002/path.5751 PMC8429220

[66] Boran C, Kandirali E, Yilmaz F, Serin E, Akyol M. Reliability of the 34βE12, keratin 5/6, p63, bcl-2, and AMACR in the diagnosis of prostate carcinoma. Urologic Oncology: Seminars Original Investigations. 2011;29(6):614–23. 20189848 10.1016/j.urolonc.2009.11.013

[67] Li Y, Giovannini S, Wang T, Fang J, Li P, Shao C, et al. p63: a crucial player in epithelial stemness regulation. Oncogene. 2023;42(46):3371–84. 37848625 10.1038/s41388-023-02859-4 PMC10638092

[68] Potterveld SK, Williamson SR, Al-Obaidy KI, Akgul M, Chan E, Giannico GA, et al. GATA3 Expression in Prostatic Adenosquamous Carcinoma: A Potential Diagnostic Pitfall. Int J Surg Pathol. 2025;33(1):85–91. 38562047 10.1177/10668969241241640

[69] Xin Z, Zhang Y, Jiang Z, Zhao L, Fan L, Wang Y, et al. Insulinoma-associated protein 1 is a novel sensitive and specific marker for small cell carcinoma of the prostate. Hum Pathol. 2018;79:151–9. 29885405 10.1016/j.humpath.2018.05.014

[70] Salu P, Reindl KM. Agr2 in cancer and beyond: unraveling its role during protein synthesis, ER stress, and as a predictive biomarker. Mol Cell Biochem. 2025;480(10):5205–19. 40471515 10.1007/s11010-025-05318-8 PMC12515224

[71] Peng YC, Levine CM, Zahid S, Wilson EL, Joyner AL. Sonic hedgehog signals to multiple prostate stromal stem cells that replenish distinct stromal subtypes during regeneration. Proc Natl Acad Sci U S A. 2013;110(51):20611–6. 24218555 10.1073/pnas.1315729110 PMC3870668

[72] Sultanov R, Mulyukina A, Zubkova O, Fedoseeva A, Bogomazova A, Klimina K, et al. TP63–TRIM29 axis regulates enhancer methylation and chromosomal instability in prostate cancer. Epigenetics Chromatin. 2024;17(1):6. 38481282 10.1186/s13072-024-00529-7 PMC10938740

[73] Ahmad F, Cherukuri MK, Choyke PL. Metabolic reprogramming in prostate cancer. Br J Cancer. 2021;125(9):1185–96. 34262149 10.1038/s41416-021-01435-5 PMC8548338

[74] Qiu Y, Wang Y, Liu J, Sun K, Horie S, Bing Z, et al. Pericytes in castration-resistant prostate cancer associated with disease progression and immunotherapy response: insights from single-cell analysis. Cancer Cell Int. 2025;25(1):200. 40462105 10.1186/s12935-025-03838-3 PMC12135593

[75] Krossa S, Andersen MK, Midtbust E, Wess M, Kiviaho A, Sharma A et al. Deep phenotyping of the prostate tumor microenvironment reveals molecular stratifiers of relapse linked to inflammatory chemokine expression and aberrant metabolism. bioRxiv. 2024:2024.05.13.593822.

[76] Qiu Y, Wang Y, Liu J, Sun K, Liu B, Hou Q. Single-cell sequencing unveils the transcriptomic landscape of castration-resistant prostate cancer-associated fibroblasts and their association with prognosis and immunotherapy response. BMC Cancer. 2025;25(1):813. 40307786 10.1186/s12885-025-14212-x PMC12044937

[77] Hirz T, Mei S, Sarkar H, Kfoury Y, Wu S, Verhoeven BM, et al. Dissecting the immune suppressive human prostate tumor microenvironment via integrated single-cell and spatial transcriptomic analyses. Nat Commun. 2023;14(1):663. 36750562 10.1038/s41467-023-36325-2 PMC9905093

[78] Boyer LA, Plath K, Zeitlinger J, Brambrink T, Medeiros LA, Lee TI, et al. Polycomb complexes repress developmental regulators in murine embryonic stem cells. Nature. 2006;441(7091):349–53. 16625203 10.1038/nature04733

[79] Nishana M, Ha C, Rodriguez-Hernaez J, Ranjbaran A, Chio E, Nora EP, et al. Defining the relative and combined contribution of CTCF and CTCFL to genomic regulation. Genome Biol. 2020;21(1):108. 32393311 10.1186/s13059-020-02024-0 PMC7212617

[80] Xu M, Zhao GN, Lv X, Liu G, Wang LY, Hao DL, et al. CTCF controls HOXA cluster silencing and mediates PRC2-repressive higher-order chromatin structure in NT2/D1 cells. Mol Cell Biol. 2014;34(20):3867–79. 25135475 10.1128/MCB.00567-14 PMC4187707

[81] Zhu WG, Srinivasan K, Dai Z, Duan W, Druhan LJ, Ding H, et al. Methylation of adjacent CpG sites affects Sp1/Sp3 binding and activity in the p21(Cip1) promoter. Mol Cell Biol. 2003;23(12):4056–65. 12773551 10.1128/MCB.23.12.4056-4065.2003 PMC156121

[82] Lu S, Jenster G, Epner DE. Androgen induction of cyclin-dependent kinase inhibitor p21 gene: role of androgen receptor and transcription factor Sp1 complex. Mol Endocrinol. 2000;14(5):753–60. 10809237 10.1210/mend.14.5.0461

[83] Urbanucci A, Barfeld SJ, Kytölä V, Itkonen HM, Coleman IM, Vodák D, et al. Androgen Receptor Deregulation Drives Bromodomain-Mediated Chromatin Alterations in Prostate Cancer. Cell Rep. 2017;19(10):2045–59. 28591577 10.1016/j.celrep.2017.05.049 PMC5675034

[84] He J, Huang Y, Liu Z, Zhao R, Liu Q, Wei L, et al. Hypomethylation of BORIS is a promising prognostic biomarker in hepatocellular carcinoma. Gene. 2017;629:29–34. 28764977 10.1016/j.gene.2017.07.077

[85] Hoivik EA, Kusonmano K, Halle MK, Berg A, Wik E, Werner HM, et al. Hypomethylation of the CTCFL/BORIS promoter and aberrant expression during endometrial cancer progression suggests a role as an Epi-driver gene. Oncotarget. 2014;5(4):1052–61. 24658009 10.18632/oncotarget.1697 PMC4011582

[86] Available from: https://raw.githack.com/bioFAM/MOFA2_tutorials/master/R_tutorials/CLL.html.

